# Supplementary material for: Causal Relationship between Aging and Anorexia Nervosa: A White-Matter-Microstructure-Mediated Mendelian Randomization Analysis
Source: Biomedicines. 2024 Aug 16;12(8):1874. doi: 10.3390/biomedicines12081874 (PMC11351342; doi:10.3390/biomedicines12081874)
Supplement: Supplementary file 1 [file biomedicines-12-01874-s001.zip › Supplementary Figure/Figure S4/Singe SNP plot of MR analysis for TL to ubm-b-1649.pdf]

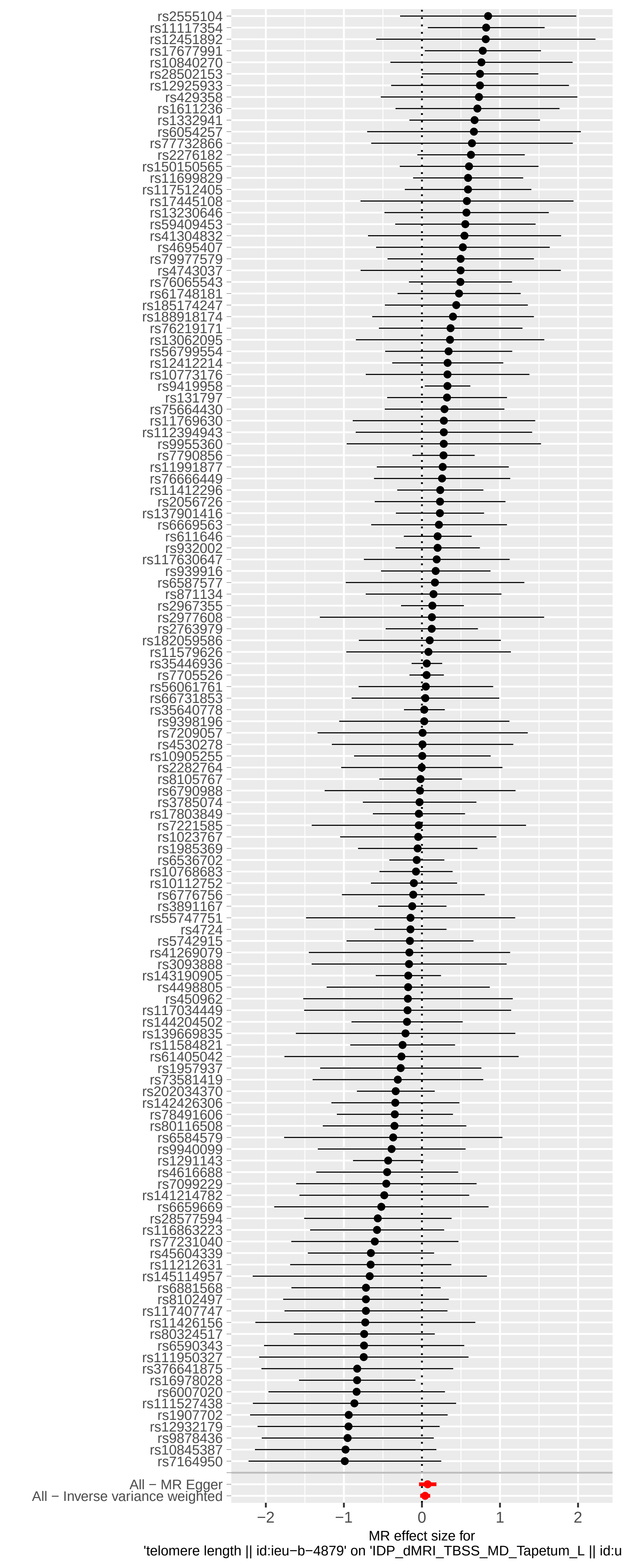
rs2555104
rs11117354
rs12451892
rs17677991
rs10840270
rs28502153
rs12925933
rs429358
rs1611236
rs1332941
rs6054257
rs77732866
rs2276182
rs150150565
rs11699829
rs117512405
rs17445108
rs13230646
rs59409453
rs41304832
rs4695407
rs79977579
rs4743037
rs76065543
rs61748181
rs185174247
rs188918174
rs76219171
rs13062095
rs56799554
rs12412214
rs10773176
rs9419958
rs131797
rs75664430
rs11769630
rs112394943
rs9955360
rs7790856
rs11991877
rs76666449
rs11412296
rs2056726
rs137901416
rs6669563
rs611646
rs932002
rs117630647
rs939916
rs6587577
rs871134
rs2967355
rs2977608
rs2763979
rs182059586
rs11579626
rs35446936
rs7705526
rs56061761
rs66731853
rs35640778
rs9398196
rs7209057
rs4530278
rs10905255
rs2282764
rs8105767
rs6790988
rs3785074
rs17803849
rs7221585
rs1023767
rs1985369
rs6536702
rs10768683
rs10112752
rs6776756
rs3891167
rs55747751
rs4724
rs5742915
rs41269079
rs3093888
rs143190905
rs4498805
rs450962
rs117034449
rs144204502
rs139669835
rs11584821
rs61405042
rs1957937
rs73581419
rs202034370
rs142426306
rs78491606
rs80116508
rs6584579
rs9940099
rs1291143
rs4616688
rs7099229
rs141214782
rs6659669
rs28577594
rs116863223
rs77231040
rs45604339
rs11212631
rs145114957
rs6881568
rs8102497
rs117407747
rs11426156
rs80324517
rs6590343
rs111950327
rs376641875
rs16978028
rs6007020
rs111527438
rs1907702
rs12932179
rs9878436
rs10845387
rs7164950
All − MR Egger
All − Inverse variance weighted
−2
−1
0
1
2
MR effect size for
'telomere length || id:ieu−b−4879' on 'IDP_dMRI_TBSS_MD_Tapetum_L || id:u
